# Early Detection of Septic Patient′s Deterioration Based on the Modified Early Obstetric Warning Score (MEOWS)—Case Report

**DOI:** 10.1155/crog/7437378

**Published:** 2026-02-15

**Authors:** Patryk Mickiewicz, Andrzej Jaworowski, Magdalena Kołak, Paweł Krawczyk, Hubert Huras

**Affiliations:** ^1^ Perinatology and Obstetrics Department, Jagiellonian University Medical College, Cracow, Poland, cm-uj.krakow.pl; ^2^ Department of Anesthesiology and Intensive Care, Jagiellonian University Medical College, Cracow, Poland, cm-uj.krakow.pl

**Keywords:** bedside assessment, maternal sepsis, MEOWS, sepsis management, urosepsis

## Abstract

**Background:**

Delayed diagnosis concerns most septic infections and results in worse prognosis. We present a case of a 35‐year‐old pregnant woman with two severe maternal infections in whom rapid implementation of MEOWS and further treatment avoided septic deterioration.

**Case Description:**

We report a case of a 26‐week gravida that was admitted due to fever, chills, muscle pain, headache, and chest pain. Clinical examination ruled out signs of preterm labor. Elevated results of implemented MEOWS prompted rapid initiation of broad‐spectrum antibiotic therapy. A microbiological examination of urine and blood confirmed *Staphylococcus aureus* MSSA. Quick implementation of the therapy allowed for the normalization of inflammatory parameters, and the patient was discharged. Three days after, she returned with similar, flu‐like signs. As with the first admission, the increase in the MEOWS score preceded the increase in inflammatory parameters. The patient went into premature labor at 30 weeks of gestation. Although histopathological examination of the placenta revealed inflammatory features, the source of infection has not been identified. Continuation of antibiotic therapy normalized inflammatory parameters, and she was discharged 8 days after the delivery.

**Conclusions:**

Delayed diagnosis is one of the most relevant factors affecting prognosis of maternal sepsis. The use of MEOWS may accelerate the decision to implement therapy and reduce maternal morbidity and mortality.

## 1. Introduction

Sepsis is a leading cause of maternal morbidity, resulting in ICU admissions [1, 2]. WHO indicates an increase in the maternal sepsis rate, causing 14% of maternal deaths in developing countries [3]. Reliable, quick, and effective bedside assessment tools, such as the modified early obstetric warning score (MEOWS), can help detect early signs of maternal deterioration. Our facility utilizes the MEOWS developed within the MACriCare project [4]. The MEOWS in use incorporates the following parameters: heart rate, blood pressure, respiratory rate, temperature, oxygen saturation, level of consciousness, lochia character, diuresis, proteinuria, and abdominal pain.

## 2. Statistical Analysis

Descriptive statistical methods were employed. Patient clinical data, laboratory values, and MEOWS scores were collected, analyzed, and graphically presented, with longitudinal changes in laboratory results depicted (Figure 1).

Figure 1Relationship between: MEOWS and level of WBC and procalcitonin during first hospitalization (a), MEOWS and level of CRP and Il‐6 during the first hospitalization (b), MEOWS and level of WBC and procalcitonin during the second hospitalization (c), MEOWS and level of CRP and Il‐6 during the second hospitalization (d).

**Figure:**
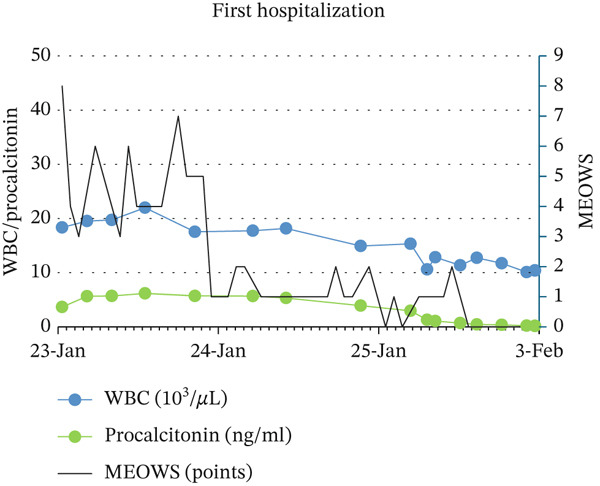
(a)

**Figure:**
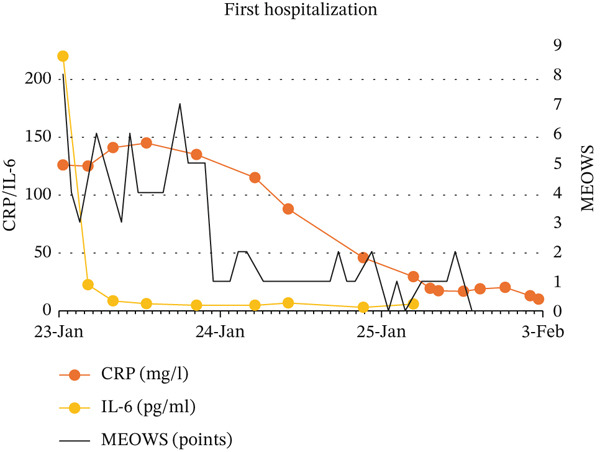
(b)

**Figure:**
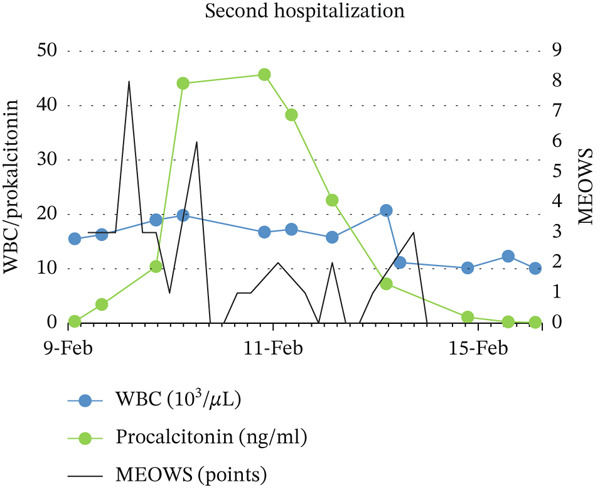
(c)

**Figure:**
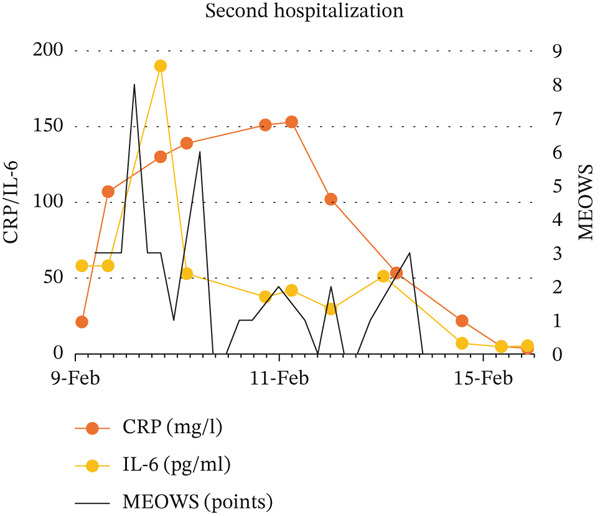
(d)

## 3. Case Report

A 35‐year‐old multigravida at 26 5/7 weeks of uneventful gestation was admitted with a 2‐day history of fever (39.5°C), chills, muscle pain, headache, and chest pain. At admission, the patient was tachypnic, temperature −37.5°C, BP 104/60 mmHg, HR 125/min, without signs of preterm labor, vaginal bleeding, or abnormal discharge. Urine output was normal, and the lactate level was low. Blood and urogenital cultures were collected, and IV fluid with piperacillin and tazobactam therapy was started. Blood test results showed white blood cells (WBC) of 18.35 × 103/*μ*L, C‐reactive protein (CRP) 126 mg/L, procalcitonin 3.66 ng/mL, interleukin 6 (IL‐6) 220 pg/mL, an increased troponin T (43.7 ng/L) and alanine transaminase (85 U/L) level. Urine and blood cultures confirmed *Staphylococcus aureus* MSSA infection, sensitive to cloxacillin. Increased MEOWS (8 points) triggered rapid response, resulting in a transfer to a higher care facility (HDU). This was followed by a significant increase in inflammatory parameters (Figure 1a,b). After excluding chorioamnionitis, the pregnant patient was discharged after 10 days of antibiotic therapy. The inflammatory parameters were normalized. At the first discharge, the provisional diagnosis was resolved methicillin‐sensitive *Staphylococcus aureus* (MSSA) bacteremia secondary to urinary tract infection.

3‐days after discharge, the patient returned with chills, fever (39°C) only (MEOWS 3 points). Laboratory tests revealed a significant rise in WBC 15.5 × 103/*μ*L, CRP 21 mg/L, procalcitonin 0.34 ng/mL, IL‐6 58.1 pg/mL, alanine transaminase (49 U/L) compared to discharge. Urine output was normal, and the lactate level was low. Empirical ceftriaxone was started after obtaining blood and genitourinary culture. Three hours later, MEOWS increased to 8 points (BP 104/42 mmHg, HR 137/min, fever). The decision was made to extend antibiotic therapy with meropenem and metronidazole. Similarly, the maximum MEOWS value anticipated the peak of inflammatory parameters (Figure 1c,d). The results of the blood culture revealed *Staphylococcus pseudintermedius*. The patient reported her husband had a staphylococcal lower limb wound infection. Performed amniocentesis revealed amniotic IL‐6 of 27104 pg/mL. The amniotic fluid culture was negative. Four days from the second admission, premature labor was present at 29 4/7 weeks of pregnancy. Histopathological examination of the placenta revealed fetal membranes with abundant neutrophil infiltration. Seven days of antibiotic therapy resulted in the normalization of inflammatory parameters. The patient′s postpartum stay was uneventful. She was discharged home 8 days after birth. At the second discharge, the provisional diagnosis was resolved chorioamnionitis secondary to *S. pseudintermedius* infection. The newborn exhibited significantly elevated inflammatory markers at birth, suggestive of early infection. Empirical antibiotic therapy with ampicillin and gentamicin resulted in prompt normalization of inflammatory parameters, with blood cultures remaining sterile after 7 days. Despite requiring extended respiratory support, the infant demonstrated normal growth and neurodevelopment at over 1 year of follow‐up.

## 4. Discussion

Delayed diagnosis of maternal sepsis is common among obstetricians [5]. In 63% of maternal sepsis, mortality was related to delays in initiation of treatment [5]. For each hour of delayed sepsis treatment, the survival risk is reduced by 7.6% [6]. Overlapping symptoms of infection and physiological changes during pregnancy make sepsis detection more challenging. Using dedicated and reliable tools to detect patients′ deterioration may help to overcome this disadvantage. Fortunately for our patient, the response triggered by MEOWS use resulted in recovery, allowing us to avoid further deterioration. In this case, MEOWS helped to quickly identify the deterioration of the patient′s condition based on objective vital signs. Objective assessment of the patient′s condition was facilitated through serial MEOWS measurements. During the initial hospitalization, the patient′s condition demonstrated stabilization within the first 24 h of commencing broad‐spectrum antibiotic therapy, evidenced by the absence of a rapid increase in the MEOWS score. By the second day, a notable improvement in clinical status was observed, with a corresponding decrease in the MEOWS score, further substantiating the positive response to treatment. While our practice had previously utilized other obstetric scales (SOS, MERC, MEWT), the value of MEOWS (89% sensitive and 79% specific, PPV 39%) is being emphasized [7]. Despite this, MEOWS was used in only 34.5% of obstetric units in the WHO Europe region, with examples of non‐obstetric scales being used [4]. This case highlights the importance of using MEOWS in early detection of maternal deterioration, including maternal sepsis. The use of MEOWS facilitated early detection of deterioration, resulting in prompt response, including transfer of the patients to a higher level of care and rapid initiation of treatment involving senior staff. MEOWS itself is not the only assessment of the patient at risk. The assessment results indicate further activity, rapid initiation of treatment, alarming senior staff, planning the next assessment time, and involving a higher level of care. However, when introducing new cognitive aids to patient management, we must be aware of the need to build awareness, offer proper training for healthcare providers, and provide resources to respond appropriately to critical illnesses. Adaptation of MEOWS on a national level improves its utilization, improving patients′ safety and resulting in decreased maternal morbidity and mortality.

## 5. Conclusions

The MEOWS enabled early recognition of maternal clinical deterioration, preceding biochemical indicators of sepsis. Its use supported timely escalation of care and initiation of targeted therapy, potentially preventing progression to severe morbidity. Routine implementation of MEOWS may enhance early detection of critical illness in obstetric patients, improve response efficiency, and contribute to better maternal outcomes.

## Author Contributions

Patryk Mickiewicz contributed to patient care, conception and design of the study, and to drafting the article. Andrzej Jaworowski contributed to patient care, data analysis and interpretation, and to critical revision of the article for important intellectual content. Magdalena Kołak contributed to patient care, data acquisition and to drafting the article. Paweł Krawczyk contributed to data acquisition and to critical revision of the article for important intellectual content. Hubert Huras contributed to data analysis and interpretation, and to critical revision of the article for important intellectual content.

## Funding

No funding was received for this manuscript.

## Disclosure

This article was not commissioned and was peer reviewed. Corresponding author, Patryk Mickiewicz, affirms that this manuscript is an honest, accurate, and transparent account of the study being reported; that no important aspects of the study have been omitted; and that any discrepancies from the study as planned (and, if relevant, registered) have been explained.

All authors have read and approved the final version of the manuscript. Patryk Mickiewicz had full access to all of the data in this study and takes complete responsibility for the integrity of the data and the accuracy of the data analysis.

## Consent

Written informed consent was obtained from the patient for the publication of this case report.

## Conflicts of Interest

The authors declare no conflicts of interest.

## Supporting information


**Supporting Information** Additional supporting information can be found online in the Supporting Information section. The supporting information contains the modified early obstetric warning score (MEOWS) chart used in our institution, detailing scoring thresholds for physiological and clinical parameters.

## Data Availability

The data that support the findings of this work are available on request from the corresponding author. The data are not publicly available due to privacy or ethical restrictions.
