# Supplementary material for: Early Detection of Septic Patient′s Deterioration Based on the Modified Early Obstetric Warning Score (MEOWS)—Case Report
Source: Case Rep Obstet Gynecol. 2026 Feb 15;2026:7437378. doi: 10.1155/crog/7437378 (PMC12907564; doi:10.1155/crog/7437378)
Supplement: Supplementary file 1 — Supporting Information Additional supporting information can be found online in the Supporting Information section. The supporting information contains the modified early obstetric warning score (MEOWS) chart used in our institution, detailing scoring thresholds for physiological and clinical parameters. [file CROG-2026-7437378-s001.docx]

Name …………………………………………………………..

ID number …………………………………………………………………………

| **Parameter** | **Modified Early Obstetric Warning Score** | | | | | | |
| --- | --- | --- | --- | --- | --- | --- | --- |
|  | **3** | **2** | **1** | **0** | **1** | **2** | **3** |
| **Respiratory Rate** | **<12** |  |  | **12-20** | **21-25** | **26-30** | **>30** |
| **Saturation** | **<92** | **92-95** |  | **>95** |  |  |  |
| **Oxygen therapy** |  | **Yes** |  | **No** |  |  |  |
| **Temperature** | **<36** |  |  | **36.1-37.2** |  | **37.3-37.7** | **>37.7** |
| **Systolic blood pressure** | **≤69** | **70-89** | **90-99** | **100-139** | **140-149** | **150-159** | **≥160** |
| **Diastolic blood pressure** |  |  | **<49** | **50-89** | **90-99** | **100-109** | **≥110** |
| **Heart rate** |  | **≤ 39** | **40-59** | **60-99** | **100-109** | **110-129** | **≥130** |
| **AVPU scale** |  | **New agitation,**  **confusion** |  | **Conscious (Alert)** | **Voice response (Voice)** | **Pain response (Pain)** | **Lack of reaction**  **(Unresponsive)** |
| **Pain (unrelated to labor)** |  |  |  | **None** |  |  | **Excessive** |
| **Lochia** |  |  |  | **Normal** |  |  | **Abnormal** |
| **Diuresis [ml/hour]** | **<10** | **<30** |  | **Not measured** |  |  |  |
| **Proteinuria** |  |  |  |  |  | **+** | **++>** |

| 0 points | Reassess in 12 hours.  In the absence of other risk factors, repeat assessment may not be required.  If in doubt, consult your resident physician or specialist.  Maintain care and documentation as usual. |
| --- | --- |
| 1-2 points | Contact your resident physician or specialist to determine reassessment frequency (every 1 or 4 or 12 hours).  Continue assessment at least once every 12 hours. |
| 3 points | Contact your resident physician or specialist.  Reassess every 4 hours.  Consider increasing the frequency of reassessment (more than 4 hours). |
| 3 points regarding one of the parameter | Assess saturation and administer oxygen if saturation < 94%.  Contact your resident physician or specialist to re-evaluate.  Re-evaluate at least once per hour.  Assess fluid balance, begin monitoring vital signs, consider diuresis monitoring (insertion of a urinary catheter).  Reassessment by physician within an hour. If not possible, call the resuscitation team.  Consider transferring the patient to a monitored room.  Inform the anesthesiologist.  Consider the risk of sepsis. |
| 4-5 points | Assess saturation and administer oxygen if saturation < 94%.  Contact your resident physician or specialist for reassessment.  Reassess at least once per hour.  Reassessment by physician within 30 minutes. If impossible, call the resuscitation team.  Consider transferring the patient to an intensive care unit/labor ward.  Inform the anesthesiologist.  Consider the risk of sepsis. |
| ≥6 points | Assess oxygen saturation and administer oxygen if saturation < 94%.  Immediately call the resident physician or specialist.  Inform the anesthesiologist.  Transfer to an intensive care unit/labor ward/calling the resuscitation team.  Assessment of vital signs at least once every 15 minutes.  Consider the risk of sepsis. |
| If consciousness is impaired, assess blood glucose levels. | |

| **Date** | | **MEOWS** |  |  |  |  |  |  |  |  |  |  |  |  |  |  |  |  |  |  |
| --- | --- | --- | --- | --- | --- | --- | --- | --- | --- | --- | --- | --- | --- | --- | --- | --- | --- | --- | --- | --- |
| **Hour** | |  |  |  |  |  |  |  |  |  |  |  |  |  |  |  |  |  |  |  |
| **Respiratory Rate** | >30 | **3** |  |  |  |  |  |  |  |  |  |  |  |  |  |  |  |  | **3** | >30 |
|  | 26-30 | **2** |  |  |  |  |  |  |  |  |  |  |  |  |  |  |  |  | **2** | 26-30 |
|  | 21-25 | **1** |  |  |  |  |  |  |  |  |  |  |  |  |  |  |  |  | **1** | 21-25 |
|  | 12-20 | **0** |  |  |  |  |  |  |  |  |  |  |  |  |  |  |  |  | **0** | 12-20 |
|  | <12 | **3** |  |  |  |  |  |  |  |  |  |  |  |  |  |  |  |  | **3** | <12 |
| **Saturation** | >95% | **0** |  |  |  |  |  |  |  |  |  |  |  |  |  |  |  |  | **0** | >95% |
|  | 92-95% | **2** |  |  |  |  |  |  |  |  |  |  |  |  |  |  |  |  | **2** | 92-95% |
|  | <92% | **3** |  |  |  |  |  |  |  |  |  |  |  |  |  |  |  |  | **3** | <92% |
| **Oxygen therapy** | YES | **2** |  |  |  |  |  |  |  |  |  |  |  |  |  |  |  |  | **2** | YES |
| **Temperature ^0^C** | >37,7 | **3** |  |  |  |  |  |  |  |  |  |  |  |  |  |  |  |  | **3** | >37,7 |
|  | 37,3-37,7 | **2** |  |  |  |  |  |  |  |  |  |  |  |  |  |  |  |  | **2** | 37,3-37,7 |
|  | 36,1-37,2 | **0** |  |  |  |  |  |  |  |  |  |  |  |  |  |  |  |  | **0** | 36,1-37,2 |
|  | <36 | **3** |  |  |  |  |  |  |  |  |  |  |  |  |  |  |  |  | **3** | <36 |
| **Systolic blood pressure** | 200 | **3** |  |  |  |  |  |  |  |  |  |  |  |  |  |  |  |  | **3** | 200 |
|  | 190 | **3** |  |  |  |  |  |  |  |  |  |  |  |  |  |  |  |  | **3** | 190 |
|  | 180 | **3** |  |  |  |  |  |  |  |  |  |  |  |  |  |  |  |  | **3** | 180 |
|  | 170 | **3** |  |  |  |  |  |  |  |  |  |  |  |  |  |  |  |  | **3** | 170 |
|  | 160 | **2** |  |  |  |  |  |  |  |  |  |  |  |  |  |  |  |  | **2** | 160 |
|  | 150 | **2** |  |  |  |  |  |  |  |  |  |  |  |  |  |  |  |  | **2** | 150 |
|  | 140 | **1** |  |  |  |  |  |  |  |  |  |  |  |  |  |  |  |  | **1** | 140 |
|  | 130 | **0** |  |  |  |  |  |  |  |  |  |  |  |  |  |  |  |  | **0** | 130 |
|  | 120 | **0** |  |  |  |  |  |  |  |  |  |  |  |  |  |  |  |  | **0** | 120 |
|  | 110 | **0** |  |  |  |  |  |  |  |  |  |  |  |  |  |  |  |  | **0** | 110 |
|  | 100 | **0** |  |  |  |  |  |  |  |  |  |  |  |  |  |  |  |  | **0** | 100 |
|  | 90 | **1** |  |  |  |  |  |  |  |  |  |  |  |  |  |  |  |  | **1** | 90 |
|  | 80 | **2** |  |  |  |  |  |  |  |  |  |  |  |  |  |  |  |  | **2** | 80 |
|  | 70 | **2** |  |  |  |  |  |  |  |  |  |  |  |  |  |  |  |  | **2** | 70 |
|  | 60 | **3** |  |  |  |  |  |  |  |  |  |  |  |  |  |  |  |  | **3** | 60 |
|  | 50 | **3** |  |  |  |  |  |  |  |  |  |  |  |  |  |  |  |  | **3** | 50 |
|  | 40 | **3** |  |  |  |  |  |  |  |  |  |  |  |  |  |  |  |  | **3** | 40 |
| **Diastolic blood pressure** | 120 | **3** |  |  |  |  |  |  |  |  |  |  |  |  |  |  |  |  | **3** | 120 |
|  | 110 | **3** |  |  |  |  |  |  |  |  |  |  |  |  |  |  |  |  | **3** | 110 |
|  | 100 | **2** |  |  |  |  |  |  |  |  |  |  |  |  |  |  |  |  | **2** | 100 |
|  | 90 | **1** |  |  |  |  |  |  |  |  |  |  |  |  |  |  |  |  | **1** | 90 |
|  | 80 | **0** |  |  |  |  |  |  |  |  |  |  |  |  |  |  |  |  | **0** | 80 |
|  | 70 | **0** |  |  |  |  |  |  |  |  |  |  |  |  |  |  |  |  | **0** | 70 |
|  | 60 | **0** |  |  |  |  |  |  |  |  |  |  |  |  |  |  |  |  | **0** | 60 |
|  | 50 | **0** |  |  |  |  |  |  |  |  |  |  |  |  |  |  |  |  | **0** | 50 |
|  | 40 | **1** |  |  |  |  |  |  |  |  |  |  |  |  |  |  |  |  | **1** | 40 |
| **Heart rate** | 140 | **3** |  |  |  |  |  |  |  |  |  |  |  |  |  |  |  |  | **3** | 140 |
|  | 130 | **3** |  |  |  |  |  |  |  |  |  |  |  |  |  |  |  |  | **3** | 130 |
|  | 120 | **2** |  |  |  |  |  |  |  |  |  |  |  |  |  |  |  |  | **2** | 120 |
|  | 110 | **2** |  |  |  |  |  |  |  |  |  |  |  |  |  |  |  |  | **2** | 110 |
|  | 100 | **1** |  |  |  |  |  |  |  |  |  |  |  |  |  |  |  |  | **1** | 100 |
|  | 60-90 | **0** |  |  |  |  |  |  |  |  |  |  |  |  |  |  |  |  | **0** | 60-90 |
|  | 50 | **1** |  |  |  |  |  |  |  |  |  |  |  |  |  |  |  |  | **1** | 50 |
|  | 40 | **2** |  |  |  |  |  |  |  |  |  |  |  |  |  |  |  |  | **2** | 40 |
| **AVPU scale** | New symptoms | **2** |  |  |  |  |  |  |  |  |  |  |  |  |  |  |  |  | **2** | New symptoms |
|  | A | **0** |  |  |  |  |  |  |  |  |  |  |  |  |  |  |  |  | **0** | A |
|  | V | **1** |  |  |  |  |  |  |  |  |  |  |  |  |  |  |  |  | **1** | V |
|  | P | **2** |  |  |  |  |  |  |  |  |  |  |  |  |  |  |  |  | **2** | P |
|  | U | **3** |  |  |  |  |  |  |  |  |  |  |  |  |  |  |  |  | **3** | U |
| **Pain scale** | None |  |  |  |  |  |  |  |  |  |  |  |  |  |  |  |  |  |  | None |
|  | Excessive | **3** |  |  |  |  |  |  |  |  |  |  |  |  |  |  |  |  | **3** | Excessive |
| **Lochia** | Normal |  |  |  |  |  |  |  |  |  |  |  |  |  |  |  |  |  |  | Normal |
|  | Abnormal | **3** |  |  |  |  |  |  |  |  |  |  |  |  |  |  |  |  | **3** | Abnormal |
| **Diuresis** | Not measured | **0** |  |  |  |  |  |  |  |  |  |  |  |  |  |  |  |  | **0** | Not measured |
|  | <30ml/h | **2** |  |  |  |  |  |  |  |  |  |  |  |  |  |  |  |  | **2** | <30ml/h |
|  | <10 ml/h | **3** |  |  |  |  |  |  |  |  |  |  |  |  |  |  |  |  | **3** | <10 ml/h |
| **Proteinuria** | + | **2** |  |  |  |  |  |  |  |  |  |  |  |  |  |  |  |  | **2** | + |
|  | ++> | **3** |  |  |  |  |  |  |  |  |  |  |  |  |  |  |  |  | **3** | ++> |
| **Glycemia level** |  |  |  |  |  |  |  |  |  |  |  |  |  |  |  |  |  |  |  | **Glycemia level** |
| **∑ MEOWS** | |  |  |  |  |  |  |  |  |  |  |  |  |  |  |  |  |  |  |  |
| **Signature** | |  |  |  |  |  |  |  |  |  |  |  |  |  |  |  |  |  |  |  |
